# The role of microRNAs in the pathogenesis of MMPi-induced skin fibrodysplasia

**DOI:** 10.1186/1471-2164-14-338

**Published:** 2013-05-20

**Authors:** Daniel P Tonge, Jonathan D Tugwood, Janet Kelsall, Timothy W Gant

**Affiliations:** 1 Centre for Radiation, Chemical and Environmental Hazards, Public Health England, Harwell Campus, Oxfordshire OX11 0RQ, UK; 2 AstraZeneca, Global Safety Assessment, Alderley Park, Macclesfield, Cheshire, UK

## Abstract

**Background:**

Matrix metalloproteinases (MMPs) are a family of proteolytic enzymes involved in extracellular matrix (ECM) homeostasis. MMPs have been an attractive pharmacological target for a number of indications. However, development has been hampered by the propensity of compounds targeting these enzymes to cause connective-tissue pathologies. The broad-spectrum MMP-inhibitor (MMPi) AZM551248 has been shown to induce such effects in the dog. Histopathological changes were consistent with fibrodysplasia (FD), characterised by fibroblast proliferation and the deposition of collagen in the subcutaneous tissues. We conducted a time-course study administering 20mg/kg/day AZM551248 between 4 and 17 days. Cervical subcutaneous tissue and plasma were sampled during the time-course. miRNA expression profiles in subcutaneous skin specimens following the administration of AZM551248 were determined by high-throughput-sequencing.

**Results:**

An increasing number of miRNAs were differentially expressed compared with vehicle treated control animals as the study progressed. Several of these were members of the miR-200 family and were significantly attenuated in response to MMPi. As the severity of FD increased at the later time-points, other miRNAs associated with TGFβ synthesis and regulation of the acute inflammatory response were modulated. Evidence indicative of epithelial to mesenchymal transition was present at all study time points. Receiver operator curve (ROC) analysis revealed that miR-21 expression in the cervical subcutaneous tissue was a sensitive and specific biomarker of FD incidence.

**Conclusions:**

Our data reveal significant perturbations in canine skin miRNA expression in response to MMPi administration. Furthermore, we have identified dysregulated miRNAs that are associated with processes relevant to the key histopathological events of MMPi-induced FD.

## Background

Matrix metalloproteinases (MMPs) are a family of approximately 30 proteolytic enzymes involved in extracellular matrix (ECM) homeostasis in normal physiology, wound-healing [1] and in various pathologies. The involvement of MMPs in pathology may be grouped into those involved in tissue destruction; for example in rheumatoid and osteoarthritis, ulceration and periodontal disease; those promoting fibrosis in fibrotic lung disease, cirrhosis and multiple sclerosis; and those that weaken the matrix as seen in cardiomyopathies and some dermatological conditions [2]. MMPs have been the subject of much investigation surrounding their potential as suitable pharmacological targets for various disease states. The first disease target of MMP inhibition was rheumatoid arthritis [2]; however in recent years matrix metalloproteinase inhibition (MMPi) has been considered for various other conditions including osteoarthritis [3], cardiovascular disease [4] and cancer [5].

Thus far, the development of many MMPis has been limited by their tendency to elicit various undesirable connective tissue pathologies in both preclinical animal models and in the clinic, at efficacious blood concentrations. In man, these side effects present as musculoskeletal syndrome (MSS), a disorder originating in the shoulder or hands, characterised by musculoskeletal pain and inflammation leading to reduced joint mobility [6]. In pre-clinical animal models, tissue pathologies tend to occur following 14 to 21 days administration of MMPi, appear to be dose-dependent, and are generally reversible on discontinuation of MMPi therapy [7]. In the rodent, clinical signs following MMPi administration include reduced movement, reluctance to rest on the hind limbs, and increased hind paw volume. Histological assessment reveals several characteristic findings including growth plate thickening, synovial hyperplasia, soft tissue fibroplasias and lymphocyte infiltrates [8]. These features associated with MMPi administration have been collectively termed "fibrodysplasia" [9]. In the canine, the most sensitive site for MMPi- induced fibrodysplasia is the subcuticular connective tissue which is affected from 11 days post-treatment, although other musculoskeletal tissues are extensively involved, as with other species, as administration continues [9].

Further compounding the liability of MMPis to elicit FD is the current lack of functional biomarkers suitable for early in vivo screening. Indeed, to date most studies have concentrated on the effects of MMPis at the end-of-treatment once FD is manifest, and following termination, using a variety of time-intensive histological techniques [6]. It is this inability to screen for early FD effects in vivo that led us to consider alternative classes of biomarkers. One class of potentially informative biomarkers is the microRNA family (miRNA). These short, non-protein-coding RNAs modulate protein translation from specific mRNAs [10]. They have been implicated in a variety of biological processes and are reported to regulate the majority of genes in the human genome [11]. Similar to many messenger RNAs (mRNA), miRNAs exhibit marked tissue specificity [12], and appear to be dysregulated in response to specific pathological conditions [10]. Perhaps most significant is the finding that miRNAs are readily detectable in various biological fluids (including plasma and serum) [13] and remain stable during routine clinical processing [14], paving the way for their use as novel biomarkers.

Herein, we report a global assessment of miRNA expression in the cervical subcutaneous tissue of dogs treated with the broad-spectrum MMPi AZM551248 at a dosage of 20mg/kg/day for between 4 and 17 days. This dose has been previously shown to induce subcutaneous FD following 17 days of administration in a pilot study involving two female beagle dogs (data not shown). A detailed histopathological report of the changes associated with FD in this study was published first in 2009 [9] and described a phased onset of FD in the cervical subcutaneous tissue following 11 days administration of AZM551248. More recently, an analysis of the changes in messenger RNA (mRNA) expression and in MMP activity following AZM551248 administration was reported [15]. The experimental aims of this study were to (I) further characterise the molecular events directly preceding the initiation and during the development of MMPi-induced FD in the same study subjects by considering miRNA dysregulation, and (II) to identify whether any miRNA(s) have the potential to serve as informative biomarkers of FD, thus addressing a current unmet need in the safety assessment of MMPis.

## Results

### In vivo histopathological analysis and plasma procollagen type III aminoterminal peptide (PIIINP) determination

The full range of histopathological changes observed in response to MMPi administration have been discussed in detail elsewhere [9]. Briefly, animals receiving vehicle or MMPi for 4 days showed no evidence of fibrodysplastic change. One animal showed evidence of fibrodysplasia following 8 days (n = 5) administration, 2 following 11 days administration (n = 5) whilst all animals showed varying severities of FD following administration of MMPi for 14 or 17 days (n = 5 respectively).

**Figure 1:**
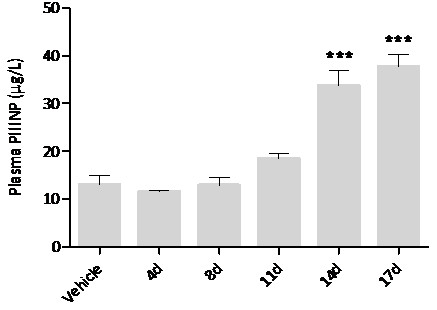
**Changes in plasma PIIINP concentration in response to AZM551248 administration.** Data are mean plasma PIIINP concentration (μg/l plasma) following administration of vehicle (control) or MMPi for 4, 8, 11, 14 and 17 days + S.E.M (n = 5). Data are analysed by one-way analysis of variance with Dunnett's post hoc test (comparing all experimental groups to vehicle) where P < 0.05; *** denotes P < 0.001.

Plasma procollagen type III aminoterminal peptide (PIIINP) has been reported previously as a prospective biomarker of various fibrotic pathologies affecting the skin [16], liver [17] and heart [18]. Plasma PIIINP concentration was unaffected by MMPi administration up to 8 days in duration when compared to vehicle treated controls. Administration of MMPi for 11 days resulted in elevated plasma PIIINP concentrations compared with vehicle treated controls (Figure 1). This elevation in plasma PIIINP concentration was coincident with the first histological evidence of FD and was maintained until the study end point at 17 days. Plasma PIIINP concentration was found to be highly correlated with the histopathological grade of FD in the cervical subcutaneous tissue (r = 0.73) as determined by the Pearson product–moment correlation coefficient (data not shown).

### Global miRNA profiling

**Figure 2:**
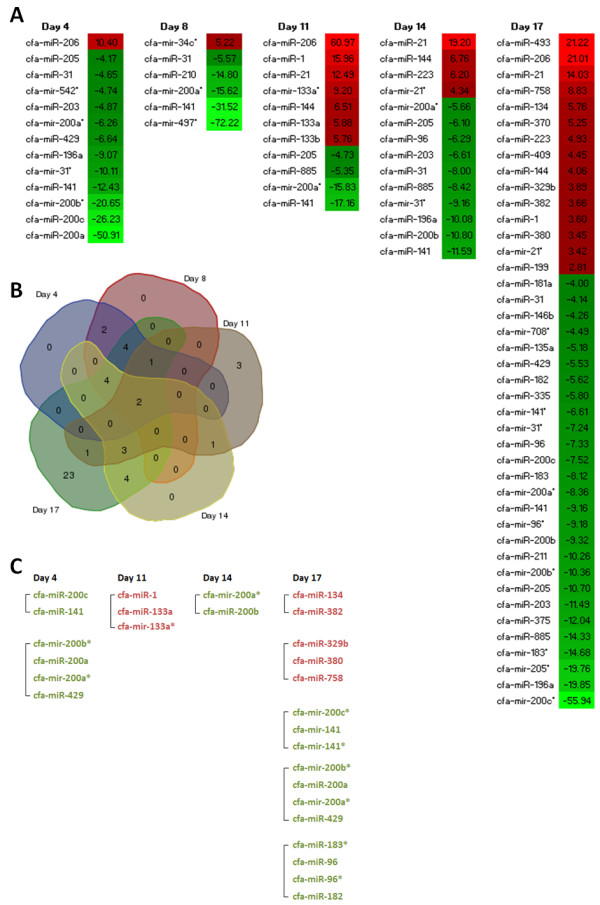
**Assessment of miRNAs differentially expressed in response to AZM551248 administration.** (**A**) Heat map generated from high-throughput sequencing data (miRNA-seq) showing miRNA expression changes in the cervical subcutaneous tissue in response to AZM551248 (n = 4 for days 4, 8, 11, 14 and 17 respectively). Data are the miRNA identifier (miRBase V16) and signed fold change relative to vehicle treated controls. Green colouration denotes significant down-regulation whilst red colouration denotes significant up-regulation. Only miRNAs were the corrected P value < 0.05 following analysis with DESeq (see materials and methods) are included within this analysis. Nomenclature: miR - mature miRNA, miR* - minor product of mature miRNA, suffix (for example a, b, c) - denotes miRNAs with closely related mature sequences. (**B**) Five-order Venn diagram demonstrating the number of miRNAs differentially regulated at each time point following AZM551248 administration. (**C**) Identification of miRNAs clustered within 10Kb of each other on the canine genome. Clusters are denoted by the inclusion of a bracket. The direction of expression change relative to vehicle treated control animals is denoted by the text colour (green down-regulated; red - up-regulated). Note the absence of any clustered miRNAs following 8 days administration of AZM551248.

**Figure 3:**
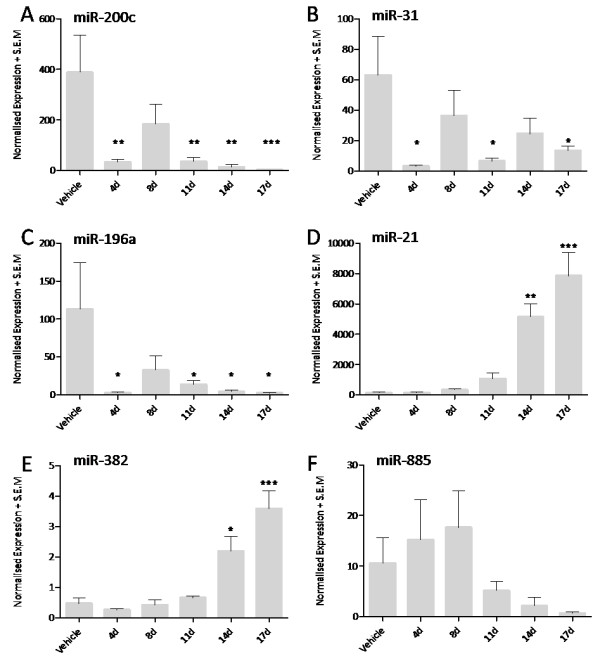
**Reverse transcriptase real-time PCR verification of expression changes in canine miRNAs miR-200c, miR-31, miR-196a, miR-21, miR-382 and miR-885 (panels A - F respectively).** Data are mean relative expression units, controlled for reverse transcription efficiency and normalised to miR-191, in cervical subcutaneous tissue following administration of vehicle (control) or MMPi for 4, 8, 11, 14 and 17 days + S.E.M (n = 5). Data are analysed by one-way analysis of variance with Dunnett's post hoc test (comparing all experimental groups to vehicle) where P < 0.05; * denotes P < 0.05, ** denotes P < 0.01 and *** denotes P < 0.001.

An average of 5,800,000 ± 400,000 total reads with an average length of 21.83 ± 0.03 bases (*mean ± standard error of the mean*) were obtained from each cervical subcutaneous lesion. Significant changes in miRNA abundance following 4, 8, 11, 14 and 17 days administration of MMPi are detailed in Figure 2A, and a comprehensive set of miRNA expression data are included within Additional file 1. The number of significantly regulated miRNAs relative to vehicle treated controls appeared to increase over time with the exception of animals treated for 4 days which exhibited more changes than those treated for 8 or 11 days (*Day 4: 13 miRNAs; Day 8: 6 miRNAs; Day 11: 11 miRNAs*). The time point(s) at which each miRNA was dysregulated are presented in the form of a 5-order Venn diagram (Figure 2B). A selection of miRNA changes were validated by reverse transcriptase real-time PCR, results of which are presented in Figure 3. In order to explore any potential relationships between the various miRNAs regulated at each time point and their potential regulatory mechanisms, results of genomic clustering analyses were obtained from miRBase V16.0. These data describe miRNAs within 10 kilo-bases (Kb) of each other on the canine genome, and therefore identify miRNAs with the potential to be transcriptionally co-regulated. Clusters of miRNAs are denoted in Figure 2C.

#### Initiating events - miRNA modulation following 4 days AZM551248 administration

Despite the lack of histopathological changes in the cervical subcutaneous tissue following 4 days administration of AZM551248, 13 miRNAs were found to be differentially expressed compared to vehicle treated control animals (Figure 2A). Of these, 7 miRNAs were found to be members of the miR-200 family - a collection of miRNAs grouped by their functional similarities and location on the genome.

**Figure 4:**
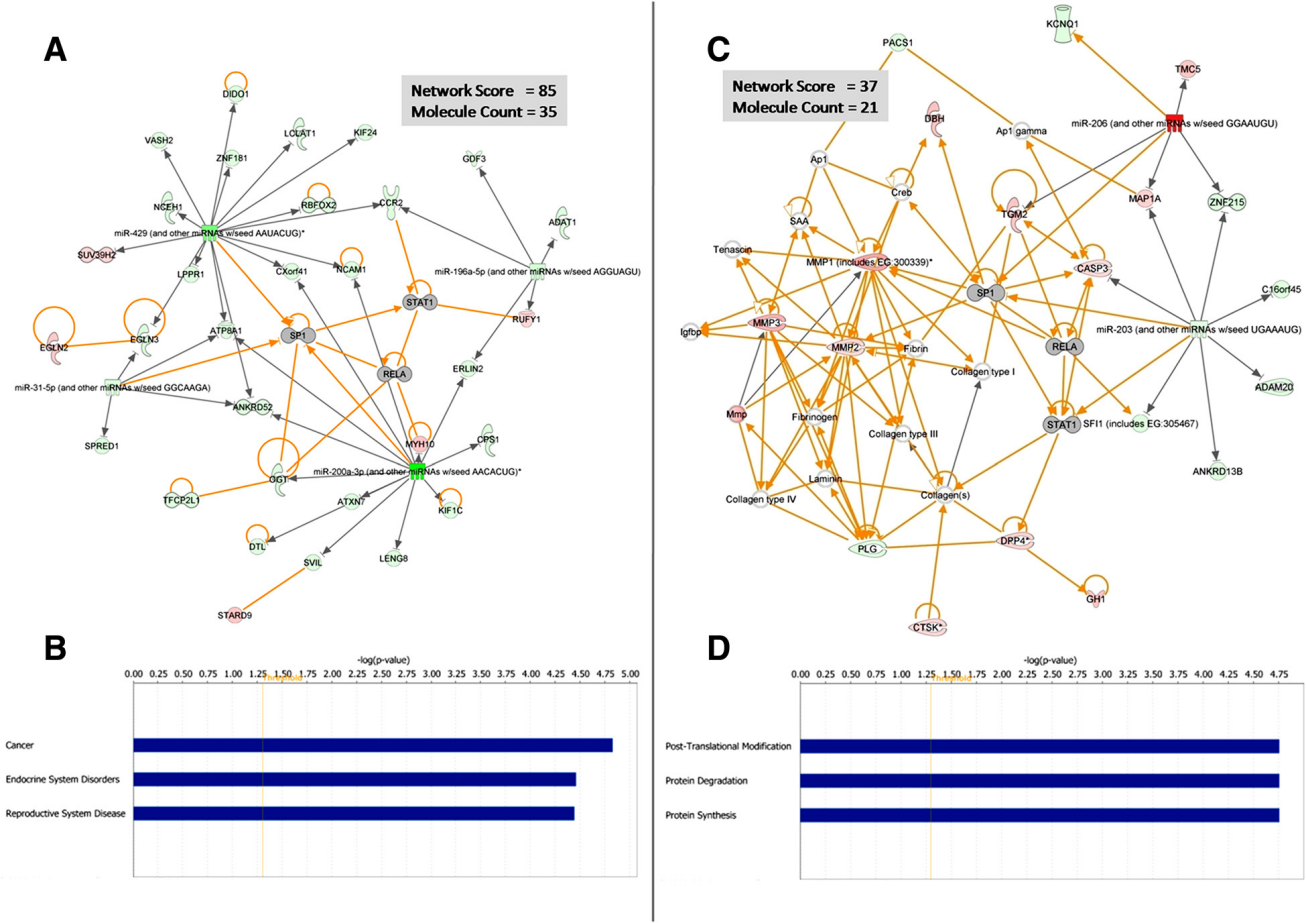
**The top two scoring gene-networks (A and C) of dysregulated miRNAs and mRNAs, and associated biological functions (B and D) in response to 4 days administration of MMPi as determined by Ingenuity Pathway Analysis.**4A and 4C - Data are mean fold change represented graphically - green colouration denotes significant down-regulation whilst red colouration denotes significant up regulation (P < 0.05). Arrows between molecules denote a stimulatory effect and blocked arrows denote a suppressive effect. Note: Transcription factors Sp1, RelA, and STAT1 are included due to their association with gene changes involved in the development of MMPi induced fibrodysplasia. Network score = −log (Fishers Exact Test result); Molecule Count refers to the number of molecules included within the network for which expression data is available (miRNA or mRNA). 4B and 4D - Data are calculated P values (−Log) determined by the Fishers Exact Test for the most significant biological functions associated with the top two scoring gene networks. Significance was accepted were P < 0.05 (−Log P > 1.301) as shown by the threshold line in orange.

In order to explore the potential role of these miRNAs in the initiation of pre-histopathological changes in response to MMPi administration, they were considered in the context of changes in the mRNA environment at this time using a concatenated dataset comprising 13 miRNAs and 221 mRNAs. The concatenated dataset was prepared by combining both mRNA and miRNA expression data (*see materials and methods section*). Previous reports have suggested that the early changes in response to MMPi administration were associated with the activation of transcription factors Sp1, RelA and STAT1 [15]. The changes in mRNA and miRNA expression were therefore presented in the context of Sp1, RelA and STAT1 activation by including these transcription factors within the pathway analysis. Functions of the top two scoring gene networks and associated gene network diagrams are presented in Figure 4 (Day 4), and Additional file 2 (Days 8–14).

Four days administration of AZM551248 was associated with regulation of gene networks involved in (1) Cancer, Endocrine System Disorders, Reproductive System Disease and (2) Post-translational Modification, Protein Degradation, Protein Synthesis. Analysis of the most significant gene regulatory networks (Figure 4) revealed a complex interplay between several messenger RNAs, transcription factors, and the dysregulated miRNAs. It is noteworthy that the top regulatory network (Figure 4A) included 4 out of the 13 differentially regulated miRNAs identified at day 4 (all 4 were down-regulated) and that there were extensive predicted interactions with the transcription factors Sp1, RelA and STAT1. The second most significant regulatory network included many factors involved in extracellular matrix remodelling including MMPs 1, 2 and 3, and the proteases dipeptidyl peptidase IV (DPP4) and Cathepsin K. Of interest was the finding that miRNAs 31, 200a*, 203 and 429 (included in Figures 4A/4C) were predicted to target Sp1 suggesting their involvement in the negative regulation of this transcriptional regulator.

#### Disease development and progression - miRNA modulation from day 8 to day 17

AZM551248 administration for a duration of 8 days saw evidence of histological changes in the cervical subcutaneous tissue consistent with FD, however this was limited to a single animal (for which sequencing data was collected), with the remaining 4 animals appearing histologically normal. In confirmation of this limited change in phenotype, this time point was associated with just 6 dysregulated miRNAs (Figure 2A), half of which were maintained from day 4 suggesting that molecular modulation at day 4 may be key to the initiation of fibrodysplasia. Novel findings at day 8 included the up-regulation of cfa-miR-34c*, and the down-regulation of cfa-miR-497* and cfa-miR-210. Combined miRNA and mRNA pathway analysis (comprising 6 miRNAs and 228 mRNAs) revealed that eight days administration of MMPi was associated with regulation of gene networks involved in (1) Post-translational Modification, Protein Degradation, Protein Synthesis (as previously identified at day 4), and (2) Hereditary Disorder, Skeletal and Muscular Disorders, Cellular Development and Cancer (Additional file 2). Both gene networks highlighted the involvement of transcription factors Sp1, RelA and STAT1. However, miRNA involvement was limited to cfa-miR-200a* which had been down-regulated previously at day 4.

AZM551248 administration for 11 to 17 days was associated with an increase in histological evidence of FD in the cervical subcutaneous tissue, and in the number of significantly dysregulated miRNAs (Figure 2A). Day 11 was associated with 11 dysregulated miRNAs of which 7 had not been dysregulated at the earlier time points, and marked the appearance of cfa-miR-21 as the most highly expressed miRNA. Clustering analysis revealed that two of these miRNAs were located within 10Kb on canine chromosome 7. Combined pathway analysis (comprising 11 miRNAs and 505 mRNAs) revealed that eleven days administration of AZM551248 was associated with the regulation of gene networks involved in (1) Cellular Development, Embryonic Development, Nervous System Development and Function, and (2) Cardiovascular Disease, Cellular Movement, Haematological System Development and Function. The most significant network highlighted an interplay between miRNAs cfa-miR-144, cfa-miR-200* and cfa-miR-885, the transcription factors Sp1, RelA and STAT1, and marked the first elevation of transforming growth factor beta transcript (TGFβ). It is interesting to note the up-regulation of cfa-miR-144 at this time point and its potential role in the regulation of TGFβ via STAT1 repression (Additional file 2).

Fourteen days administration of AZM551248 was associated with 14 dysregulated miRNAs compared with vehicle treated controls. However, only three of these dysregulated miRNAs were novel changes. This time point was also associated with the significant up-regulation of miR-21* (the minor product). Pathway analysis (comprising 14 miRNAs and 2509 mRNAs) revealed the most significant changes to be associated with (1) Post-translational Modification, Protein Folding, Cell Cycle and (2) Connective Tissue Disorders, Dermatological Diseases and Conditions. As with previous time points, the transcription factors SP1, RelA and STAT1 were shown to interact extensively with these gene networks, but miRNA involvement was not evident (Additional file 2).

Seventeen days administration of AZM551248 was associated with 42 dysregulated miRNAs compared with vehicle treated controls, of these 23 were novel changes. Of particular interest were pro-fibrotic miRNAs miR-199 and miR-382 which were significantly up-regulated (Figure 2A), and anti-inflammatory miR-335 which was significantly down-regulated. Pathway analysis (comprising 42 miRNAs and 2947 mRNAs) revealed the most significant differential gene regulation to be associated with (1) Amino Acid Metabolism, Cell Morphology, Cellular Compromise and (2) Skeletal and Muscular System Development, Cell to Cell Signalling (Additional file 2). These gene networks centred on miRNAs cfa-miR-203 and cfa-miR-200a* respectively, highlighting a variety of interactions between these miRNAs and the dysregulated mRNAs identified previously. It is interesting to note that Sp1, RelA and STAT1 involvement was absent at this time point (within the most significant gene networks) (Additional file 2) suggesting that the role of these transcription factors may be related to the initiating events in fibrodysplasia rather than disease progression.

#### Candidate tissue-based miRNA biomarkers of cervical subcutaneous Fibrodysplasia

In addition to revealing the interesting possibility that miRNAs may be involved in the initiation and progression of fibrodysplastic changes, the potential of these small non-coding RNAs to function as tissue-based biomarkers of the incidence or severity of such changes was also investigated. Raw sequencing read counts for each animal were normalised as previously described and compared with both the histological score and plasma PIIINP concentration (see Figure 1). Only miRNAs previously identified as significantly regulated were included in this analysis (Figure 2A). Correlation with plasma PIIINP concentration was used to filter the most significant associations given that this data was continuous in nature (as opposed to the discrete histological scores), and has been show to correlate highly with the histopathological findings (r = 0.73).

MicroRNAs cfa-miR-382, cfa-miR-380, cfa-miR-199, cfa-miR-223 and cfa-miR-21 (including its less abundant product cfa-miR-21*) were most strongly positively associated with plasma PIIINP concentration (r = 0.53, 0.50, 0.49, 0.47, and 0.46 respectively). It is interesting to note that cfa-miR-21 and cfa-miR-21* were significantly elevated as early as day 11 at which point the most significant pathological changes first became apparent, and increased in expression as time progressed (and the mean histopathological score and plasma PIIINP concentration increased). All other positively correlated miRNAs were only significantly elevated from day 14 (cfa-miR-223) or day 17 (cfa-miR-382, cfa-miR-380 and cfa-miR-199) despite appearing to be more closely associated with plasma PIIINP concentration.

Moderate inverse correlations with plasma PIIINP concentration were found with microRNAs cfa-miR-885, cfa-miR-375, cfa-miR-211, cfa-miR-200c*, and cfa-miR-182 (r = −0.36, -0.34, -0.27, -0.25, and −0.25 respectively). MicroRNA cfa-miR-885 expression was significantly decreased from 11 days whereas the remainder of these miRNAs were first significantly decreased at day 17 (Figure 2A). Results of the 10 most highly correlated miRNAs are included within Table 1.

**Table 1:** Correlation of miRNA expression, plasma PIIINP concentration and histological evidence of FD

**miRNA**	**Correlation with Plasma PIIINP concentration (r)**	**Correlation with histopathological score (r)**	**First detection of significant regulation (Study Day)**
cfa-miR-382	0.53	0.28	Day 17
cfa-miR-380	0.50	0.29	Day 17
cfa-miR-199	0.49	0.25	Day 17
cfa-miR-223	0.47	0.31	Day 14
cfa-miR-21	0.46	0.43	Day 11
cfa-miR-885	−0.36	−0.52	Day 11
cfa-miR-375	−0.34	−0.48	Day 17
cfa-miR-211	−0.27	−0.47	Day 17
cfa-miR-200c*	−0.25	−0.42	Day 17
cfa-miR-182	−0.25	−0.50	Day 17

Correlation of miRNA expression in the cervical subcutaneous tissue with plasma PIIINP concentration and histological evidence of fibrodysplasia. Data are r values for the top 10 most highly correlated miRNAs. This analysis was limited to those miRNAs previously identified as being significantly differentially expressed by global miRNA sequencing where P< 0.05 following FDR correction (N = 24).

**Figure 5:**
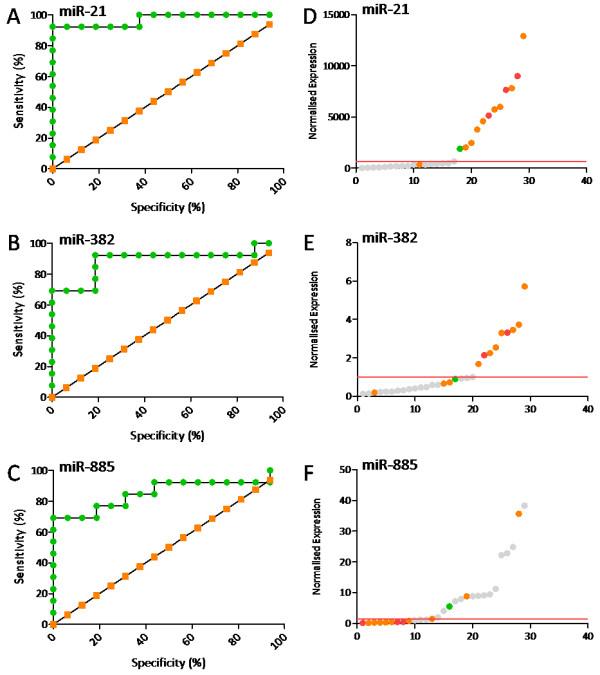
**Receiver operator curve analysis of candidate biomarkers miR 21, miR-382 and miR-885 (panels A - C respectively).** Data are normalised expression values generated by duplicate miRNA PCR reactions, controlled for reverse transcription efficiency and normalised to miR-191. Animals were grouped into those without any histological evidence of FD (n = 16) and those with histological evidence of FD at any grade (n = 13). MicroRNA-21 (miR-21) was predictive of FD incidence with a sensitivity of 92.31% and specificity of 93.75% (AUC = 0.97). MicroRNAs-382 (miR-382) and miR-885 revealed similar predictive abilities with sensitivity and specificity scores of 69.23% and 93.75% respectively (AUC = 0.89 and 0.86 respectively). Sensitivity and specificity are displayed graphically by the green line. The orange line denotes the line of identity which highlights the shape of the curve should the biomarker offer no discrimination between control and cases. Panels **D** - **F** display corresponding expression values for each individual animal ranked from the lowest to the highest expression value. Incidence and severity of FD in the cervical subcutaneous tissue is denoted by the colour of each data point; Grey Circles - no evidence of FD, Green Circles - mild FD; Orange Circles - moderate FD; Red Circles - severe FD. The cut-off value at which maximum sensitivity and specificity is obtained for each biomarker is marked by a red bar (above threshold for miRs 21 and 382, and below threshold for miR-885).

In order to further validate the potential of the identified miRNAs as novel tissue-based biomarkers of FD, reverse transcriptase real-time miRNA-PCR assays were obtained for candidate biomarkers miR-21, miR-382 and miR-885. These were selected on the basis of their high level of correlation with evidence of FD, and the availability of experimentally validated assays for the canine. Receiver operator curve (ROC) analysis was performed and area under the curve (AUC) values computed to ascertain the ability of each prospective biomarker to correctly identify diseased and disease-free animals [19]. The ability of these potential biomarkers to discriminate between the various severities of FD was not examined due to the limited number of cases in each category. MicroRNA-21 (miR-21) expression showed the highest level of discrimination with an AUC value of 0.97, whilst microRNAs miR-382 and miR-885 reported AUC values of 0.89 and 0.86 respectively. As a benchmark, the classical biomarker PIIINP reported an AUC value of 0.91. Results of the ROC analysis are presented in Figure 5 (panels A - C) and ranked expression values of each biomarker and histopathological grade presented in (panels D - F) with the suggested cut-off values denoted by a red line.

## Discussion

The purpose of this study was to further explore the molecular events surrounding the initiation and progression of AZM551248-induced fibrodysplasia (FD) in the canine. A detailed histopathological report of the changes associated with FD in this study was published first in 2009 [9] and described a phased onset of FD in the cervical subcutaneous tissue following 11 days administration of AZM551248. More recently, an analysis of the changes in messenger RNA (mRNA) expression and in MMP activity following AZM551248 administration was published [15]. The current manuscript builds upon previous work by investigating the role of microRNAs (miRNAs) during the initiation of FD and furthermore, considers their potential as novel tissue-based biomarkers of FD.

Of the 13 miRNAs dysregulated at study day 4, 7 were found to be members of the miR-200 family which has been extensively investigated and its attenuation in expression implicated in the transition of epithelial cells to a mesenchymal phenotype [20] in various mammalian systems. Epithelial to mesenchymal transition (EMT) is a biological process that allows epithelial cells to undergo various biochemical changes to assume the characteristics of a mesenchymal cell [21]. EMT is categorised into three separate processes depending on when it occurs; type I EMT occurs during embryogenesis and mediates the transition from primitive epithelia to a mesenchymal phenotype, type II is associated with organ fibrosis [22,23] and mediates the transition from epithelial cells to fibroblasts [24], whilst type III is linked with cancer progression and metastasis [21]. Given the histological findings reported, and the significant down-regulation of all members of the miR-200 family it is plausible that MMPi administration induces type II EMT; the molecular signature of which is apparent from just 4 days administration. Attenuation of miR-200 family members has been reported in various fibrotic pathologies including those of the lung ([23], kidney [25] and gastrointestinal tract [26]. Eight days of MMPi administration was associated with limited histological changes and this was reflected by the appearance of just two new miRNA perturbations, both of which corresponded to the minor (star) products of miR-34c and miR-497 respectively.

Eleven days administration of AZM551248 was associated with the first histological evidence of FD and also marked the first appearance of elevated TGFβ transcript, consistent with the detection of immuno-reactive TGFβ in activated fibroblasts [9], and in the plasma [15]. Day 11 marked the appearance of cfa-miR-21 as the most highly expressed miRNA. Prior to this time point, miR-145 was detected as the most abundant miRNA in the cervical subcutaneous tissue (however its expression remained unchanged by treatment). MicroRNA-145 (miR-145) expression in the dermal tissues is associated with the regulation of melanogenesis [27]. MicroRNAs miR-133a/b have been experimentally verified as partaking in the negative regulation of TGFβ and connective tissue growth factor (CTGF) signalling during fibrosis [28,29] and are therefore reported as "anti-fibrotic" factors. Conversely, miR-21 is reported to have the opposite effect, stimulating TGFβ signalling by both the canonical and non canonical routes [30]. On balance, it appears that miR-133a/b and miR-21 act co-operatively to regulate TGFβ signalling, with the drive for positive regulation of TGFβ signalling by miR-21 overcoming the negative regulation by miR-133a/b at this time. The significant elevation of miR-21, and its apparent increase coincident with the progression of FD also adds further weight to the hypothesis that MMPi induces epithelial to mesenchymal transition. The expression of miR-21 has been reported previously as an acquired marker of EMT [24] and tissue miR-21 expression was in fact identified as a sensitive and specific biomarker of fibrodysplastic change in this study (Figure 5). Moreover, two further transcriptional regulators indicative of EMT, Snail and Twist [24], were also elevated at the transcript level in these study animals (> 2 fold, P < 0.05 from day 14) [15].

At day 14, dysregulation of three novel miRNAs was noted. MicroRNA-223 (miR-223) has been reported to partake in the regulation of the acute inflammatory response [31,32] which is pertinent given that marked inflammatory cell infiltration was reported in the histological findings around this study time point. The down-regulation of miR-203 is also of interest at this time given its repression is reported in several models of EMT [33]. Finally, this time point was also associated with the significant up-regulation of miR-21* (the minor product), suggesting marked transcription of the miR-21 pre-miRNA transcript. Seventeen days administration of AZM551248 was associated with the up-regulation of pro-fibrotic miRNAs miR-199 and miR-382 (Figure 2A), and down-regulation of anti-inflammatory miR-335. MicroRNA miR-199 is reported to induce extracellular matrix synthesis via stimulation of the calcineurin signalling pathway [34,35], whilst miR-382 is reported to induce EMT by decreasing protection against mitochondrial oxidative stress [36]. Again, these miRNA changes appear to support the hypothesis of AZM551248-induced EMT, and the histological findings of increased ECM production.

**Figure 6:**
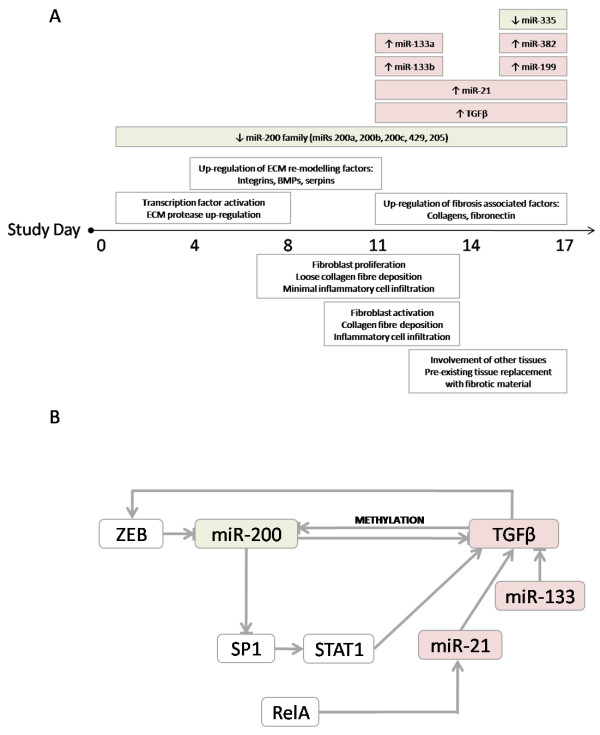
**Summary of the main histopathological and signalling events associated with AZM551248 administration.** (**A**) Summary of the main events associated with MMPi administration. Histopathological findings are shown below the bar, whilst important changes in signalling pathways and miRNA expression are shown above the bar. (**B**) The cyclic relationship between miR-200 attenuation, transcription factor activation and TGFβ production. Up-regulated factors are denoted in red, down-regulated factors in green, whilst factors not directly measured in this study appear in white. Note: below the bar adapted from (Tugwood, Kelsall et al. 2012).

Considering all of the available data, it is apparent that several pro-EMT factors including growth factors, transcriptional regulators and miRNAs are modulated from as early as 4 days administration of the broad spectrum MMPi AZM551248. These appear to orchestrate a sequence of signalling events resulting in the progressive development of FD. The following hypothesis is presented schematically in Figure 6. Around the same time as the transcription factors Sp1, STAT1 and RelA (and the proteases DPP4 and Cathepsin K) were found to be up-regulated (day 4), significant down-regulation of the miR-200 family was present. Transcription factor Sp1 is predicted to be directly targeted by the miR-200 family, suggesting that attenuation of the miR-200 family may directly contribute to the up-regulation of Sp1 and STAT1 (in-directly via Sp1). These molecular changes may also offer some explanation for the increase in TGFβ noted in response to MMPi administration. It has been shown that TGFβ-2 is directly targeted by miR-141/miR-200a, suggesting that attenuation of the miR-200 family may induce TGFβ signalling [37] through reduced inhibition. This loss of miRNA mediated repression would be in addition to the stimulation of TGFβ by STAT1. As MMPi progressed, miR-21 expression increased dramatically, the first significant changes being present from 11 days administration. MicroRNA-21 (miR-21) has been found to stimulate both the canonical and non-canonical TGFβ signalling pathways, offering a third route of TGFβ stimulation [34,35].

Given the apparent significance of miR-200 family attenuation following 4 days administration of MMPi, we sought clarity on how this family were themselves transcriptionally regulated. The transcription factor ZEB1 has been shown to repress miR-200c/141, and miR-200b/200a/429 promoter regions in several cancers [38], at which time an inverse relationship between miR-200 and ZEB expression was noted. Moreover, BMPs and TGFβ (induced in this study) are reported to activate ZEB transcription factors [39] forming a TGFβ/ZEB/miR-200 signalling network Figure 6B. Of particular interest is the finding that continued expression of TGFβ leads to the hypermethylation of miR-200 family promoter regions. This hypermethylation is found to increase with TGFβ exposure, and results in the attenuation of miR-200 expression [40]. This finding presents a further, ZEB independent method by which prolonged TGFβ expression may be self perpetuated via miR-200 family attenuation.

Moving forward, we highlight the need to further investigate the TGFβ/ZEB/miR-200 signalling network, and the specific interactions between miRNAs and the various transcription factors discussed herein (SP1, RelA and STAT1). To further study the TGFβ/ZEB/miR-200 signalling network, we propose a series of in vitro experiments conducted in an appropriate skin cell line. The proposed system would permit the over expression of TGFβ and/or various miR-200 family members (either independently or as a family) and would be amenable to methylation assessment. To further elucidate the interactions between miRNAs and the major transcription factors, we propose an additional in vitro experiment in which individual miRNAs could be over expressed and the resulting effects on both transcription factor mRNA and protein determined.

## Conclusions

We have shown that significant attenuation of the miR-200 family is noted in response to MMPi administration in the canine. Using global mRNA and miRNA data, we present a working hypothesis whereby attenuation of miR-200 contributes to the activation of several transcription factors including Sp1, STAT1 and RelA. Using pathway analysis, we have suggested a link between the attenuation of the miR-200 family, and the appearance of TGFβ at the transcript and protein levels from day 11. Furthermore, we have highlighted a potential route for maintaining this TGFβ up-regulation via a TGFβ/ZEB/miR-200 signalling network, and by the *de novo* reversible hypermethylation of the miR-200 family promoter region.

## Methods

### *AZM551248* In vivo *study*

The AZM551248 time-course study design and resulting histopathological changes have been reported in detail elsewhere [9,15]. Briefly, 30 female beagle dogs, around 12 months of age were randomly assigned to six experimental groups as described in Table 2. Animals were dosed orally once daily either with vehicle alone (0.5%w/v hydroxypropyl methylcellose/0.1% polysorbate 80, 5ml/kg), or with vehicle plus 20mg/kg/day AZM551248 [15]. Following termination, a histopathological analysis was carried out on subcutaneous tissue, dermal skin, synovium, tendon and muscle from several sites. In addition, dermal and subcutaneous tissue samples from the left and right abdomen, left and right dorsal cervical and lumbar regions, and adjacent to the calcaneal tendon and gastrocnemius muscle with associated tendon were also taken. These samples were snap frozen in liquid nitrogen for RNA analysis; the analysis described herein is confined to the dorsal cervical subcutaneous skin. Details of the histopathological analysis are described in detail elsewhere [9]. Animal experiments underwent local ethical review and were conducted in full compliance with UK Home Office guidelines.

**Table 2:** Overview of the AZM551248 study design

**Group**	**Animal Number**	**Treatment**	**Duration of Treatment (days)**	**Daily Dose (mg/kg/day)**
1	1–5	Vehicle	17	0
2	6–10	AZM551248	4	20
3	11–15	AZM551248	8	20
4	16–20	AZM551248	11	20
5	21–25	AZM551248	14	20
6	26–30	AZM551248	17	20

### Determination of plasma PIIINP concentration

Whole blood was drawn into EDTA containing tubes and plasma prepared by centrifugation. Samples were drawn on the day of termination at stored at −80°C prior to analysis. Canine plasma PIIINP was determined in duplicate by radioimmunoassay (Orion Diagnostica) against a human PIIINP standard curve supplied in the kit.

### RNA preparation

Total RNA was isolated from dorsal cervical subcutaneous skin tissue using the miRNeasy extraction kit (Qiagen), following the manufacturers standard protocol. RNA was quantitated using a NanoDrop ND-1000 spectrophotometer (Thermo Fisher) and quality was assessed using the Agilent RNA 6000 Nano Kit (Agilent). In order to enrich for small RNA molecules, total RNA samples were passed through PureLink miRNA extraction columns (Invitrogen) according to the manufacturers standard instructions. The resulting enriched RNA samples were then assessed using the Agilent Small RNA Kit (Agilent) and the percentage of small RNA (10–40 nucleotides) determined relative to the mass of total RNA.

### Preparation of SOLiD sequencing libraries

Global miRNA expression profiling of the cervical subcutaneous tissue of four randomly selected animals from each experimental group (n = 4, N = 24) was performed by next-generation sequencing using the SOLiD 4.0 system (Applied Biosystems). Briefly, 5ng of enriched RNA was ligated with SOLiD sequencing adapters overnight and reverse transcribed to synthesise complementary DNA (cDNA). The resulting cDNA was resolved on 10% v/v TBE-urea gels against a 10bp ladder (Invitrogen) and the 60-80nt region, containing miRNA-sized RNA species with adapters, excised. Gel slices were amplified by in-gel PCR using a common 5’ primer (SOLiD 5'), and a specific 3’ primer (SOLiD Barcodes 1–24) to incorporate a molecular barcode into each sample so as to permit sample multiplexing. Equimolar amounts of each library were pooled at this stage, and template preparation performed using the SOLiD EZ-Bead system and E80 emulsion PCR reagents (PN 4452722). Sequencing was performing in-house using 35 base pair (bp) chemistry (Applied Biosystems) and raw sequencing read counts were mapped to miRBase V16.0 (*Canis familiaris*), normalised to account for the varying sequencing depths between samples, and differential expression inferred using the DESeq script for R statistical language.

### Bioinformatic analysis

Primary processing of the raw sequencing data to remove adapter sequences and de-convolute the 24 multiplexed samples was undertaken during the sequencing process using BioScope software (Applied Biosystems). Following primary processing, raw sequencing reads were exported in colour-space fasta (csfasta) format into CLC Genomics Workbench (CLC). An "extract and count" routine was utilised to condense the millions of sequencing reads into a tally table of each nucleotide sequence, and the number of times that particular sequence was encountered. A separate tally table was produced for each of the 24 animals. Reads shorter than 15 nucleotides and longer than 35 nucleotides were filtered at this stage. Following parsing to remove superfluous data columns, the resulting tally tables comprising the miRNA name and count were imported into miRanalyzer V0.2 [41]. Reads were mapped to the Canis familiaris genome (CanFam2) and to miRBase version 16.0 permitting one mismatch between the sequencing reads and each index. Reads mapping to known miRNAs were counted and a relative expression value determined by dividing the number of reads mapping to each particular miRNA by the total number of reads mapped. Differential expression and P value estimation was performed using the DESeq package for R statistical language which models count-based data with negative binomial distributions and uses the method of Benjamini and Hochberg to control for type I error [42].

### Pathway analysis

Biological interpretation of the dysregulated miRNAs at each time point was undertaken using the Ingenuity Pathway Analysis (Ingenuity Systems) database accessed on June 2012. miRNAs were identified by their official sequence names (for example cfa-miR-21 for canine mature microRNA 21) and regulation was identified by the fold change values provided by DESeq. Only miRNAs with estimated P values of <0.05 following false discovery rate correction were included within each analysis. For the integrated analysis of miRNA and messenger RNA (mRNA) expression data, lists of differentially expressed mRNAs previously published by Tugwood and colleagues were utilised. mRNAs were identified by their official gene symbol, and only genes with fold changes > 2 and where P < 0.05 (students two-tailed *t*-test) were included in the analysis. To prepare the concatenated miRNA/mRNA input files for IPA, the two sources of significantly regulated molecules (miRNAs and mRNAs) were combined into a standard text file (.txt) using Excel 2007 (Microsoft). Fields included the gene identifier (official gene symbol for mRNA and official sequence name for miRNAs) and the signed fold change. A comprehensive list of gene expression changes at each time point are included within Tugwood et al. [15].

### Quantitative PCR analysis

Complementary DNA was synthesised from 1μg total RNA using the qScript miRNA cDNA synthesis kit (QuantaBio) in a total reaction volume of 20μl. Prior to reverse transcription, samples were spiked with 5nM synthetic miRNA (cel-miR-39-3p) to control for varying reverse transcription efficiencies. Each PCR reaction consisted of 5μl SYBR Green master mix (QuantaBio), 0.2μl miRNA Assay (Integrated DNA Technologies), 0.2μl Universal PCR Primer (QuantaBio), 2.6μl water and 2μl template cDNA (diluted 1:100 in 1 x TE). PCR was performed using a RotorGene Q thermocycler (Qiagen) with an initial denaturation step at 95°C for 2 minutes followed by 40 cycles of 95°C 5 seconds, 60°C 15 seconds and 70°C 15 seconds. The following miRNA Assays sourced from Integrated DNA Technologies were utilised herein (HSMIR-0031, HSMIR-0196A, HSMIR-0141, HSMIR-0885, MMIR-382*, HSMIR-0021, HSMIR-0191, HSMIR-0200C). Assay compatibility with the canine was validated by comparing the miRNA assay primer sequences with the respective canine miRNA sequence using publically available miRNA data (miRBase Version 16). All PCR data was normalised to the median expression of synthetic miRNA cel-miR-39-3p to control for reverse transcription efficiency [43] and then normalised to miR-191 which was found to be invariant across the various time points (there were no statistically significant changes between the experimental groups as determined by one-way analysis of variance).

### Statistical analyses

*Analysis of PCR Data* - all normalised PCR data were analysed by one-way analysis of variance with Dunnett's post hoc test (comparing all experimental groups to vehicle) performed where P < 0.05.

*Correlation Analysis* - assessment of correlation between the significantly regulated miRNAs (as determined by miRNA-seq) and plasma PIIINP concentration was determined by the method of Pearson using GraphPad 5.0 software (Prism) (N = 24).

*Receiver Operator Curve Analysis* - ROC analysis was performed using normalised miRNA data generated by validated canine PCR primers. ROC analysis was performed on data from all study time points and area under the curve (AUC) values were computed using GraphPad 5.0 software (Prism) as an index of discriminatory power. An AUC value of 0.5 denotes no ability to correctly identify diseased and disease-free subjects, whilst an AUC value of 1.0 denotes perfect decimation between the two groups of interest [19].

*Assessment of Significant Gene Networks* - networks of molecules identified by IPA were ranked based upon their respective "Network Score" as defined as the -log Fishers Exact Test result. Network scores are included within all gene network diagrams.

### Supporting data

A comprehensive list of all miRNAs modulated in response to AZM551248 are presented in the supplementary file (Additional file 1*.xls*).

The top two gene-networks of dysregulated miRNAs and mRNAs in response to 8, 11, 14 and 17 days administration of MMPi are presented in the supplementary file (Additional file 2*.pdf*).

## Competing interests

JDT and JK were employees of AstraZeneca, Alderley Park at the time of manuscript preparation. The authors declare no further competing interests.

## Authors’ contributions

DPT performed the molecular biology, bioinformatics and developed the integrated miRNA/mRNA analysis. JK performed the plasma PIIINP determination. All authors read and approved the final manuscript.

## Supplementary Material

Additional file 1 A comprehensive list of all miRNAs modulated in response to AZM551248 administration as determined by miRNA-seq. Click here for file

Additional file 2 The top two scoring gene-networks of miRNAs and mRNAs dysregulated in response to 8, 11, 14 and 17 days administration of AZM551248. Click here for file
