# Supplementary material for: The role of microRNAs in the pathogenesis of MMPi-induced skin fibrodysplasia
Source: BMC Genomics. 2013 May 20;14:338. doi: 10.1186/1471-2164-14-338 (PMC3668254; doi:10.1186/1471-2164-14-338)
Supplement: Additional file 2 — The top two scoring gene-networks of miRNAs and mRNAs dysregulated in response to 8, 11, 14 and 17 days administration of AZM551248. [file 1471-2164-14-338-S2.pptx]

## Slide 1
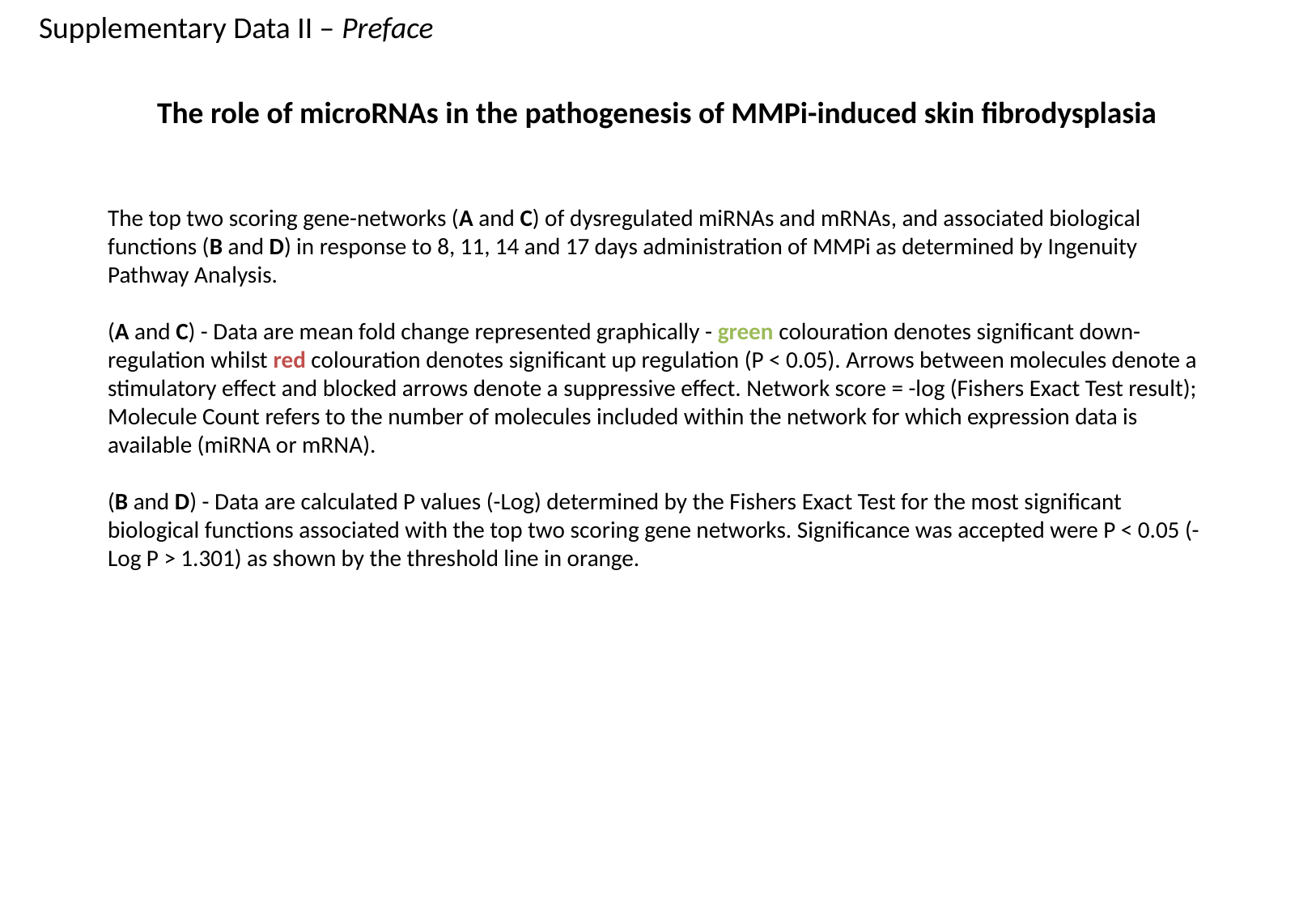

Supplementary Data II – Preface
The role of microRNAs in the pathogenesis of MMPi-induced skin fibrodysplasia
The top two scoring gene-networks (A and C) of dysregulated miRNAs and mRNAs, and associated biological functions (B and D) in response to 8, 11, 14 and 17 days administration of MMPi as determined by Ingenuity Pathway Analysis.
(A and C) - Data are mean fold change represented graphically - green colouration denotes significant down-regulation whilst red colouration denotes significant up regulation (P < 0.05). Arrows between molecules denote a stimulatory effect and blocked arrows denote a suppressive effect. Network score = -log (Fishers Exact Test result); Molecule Count refers to the number of molecules included within the network for which expression data is available (miRNA or mRNA).
(B and D) - Data are calculated P values (-Log) determined by the Fishers Exact Test for the most significant biological functions associated with the top two scoring gene networks. Significance was accepted were P < 0.05 (-Log P > 1.301) as shown by the threshold line in orange.

## Slide 2
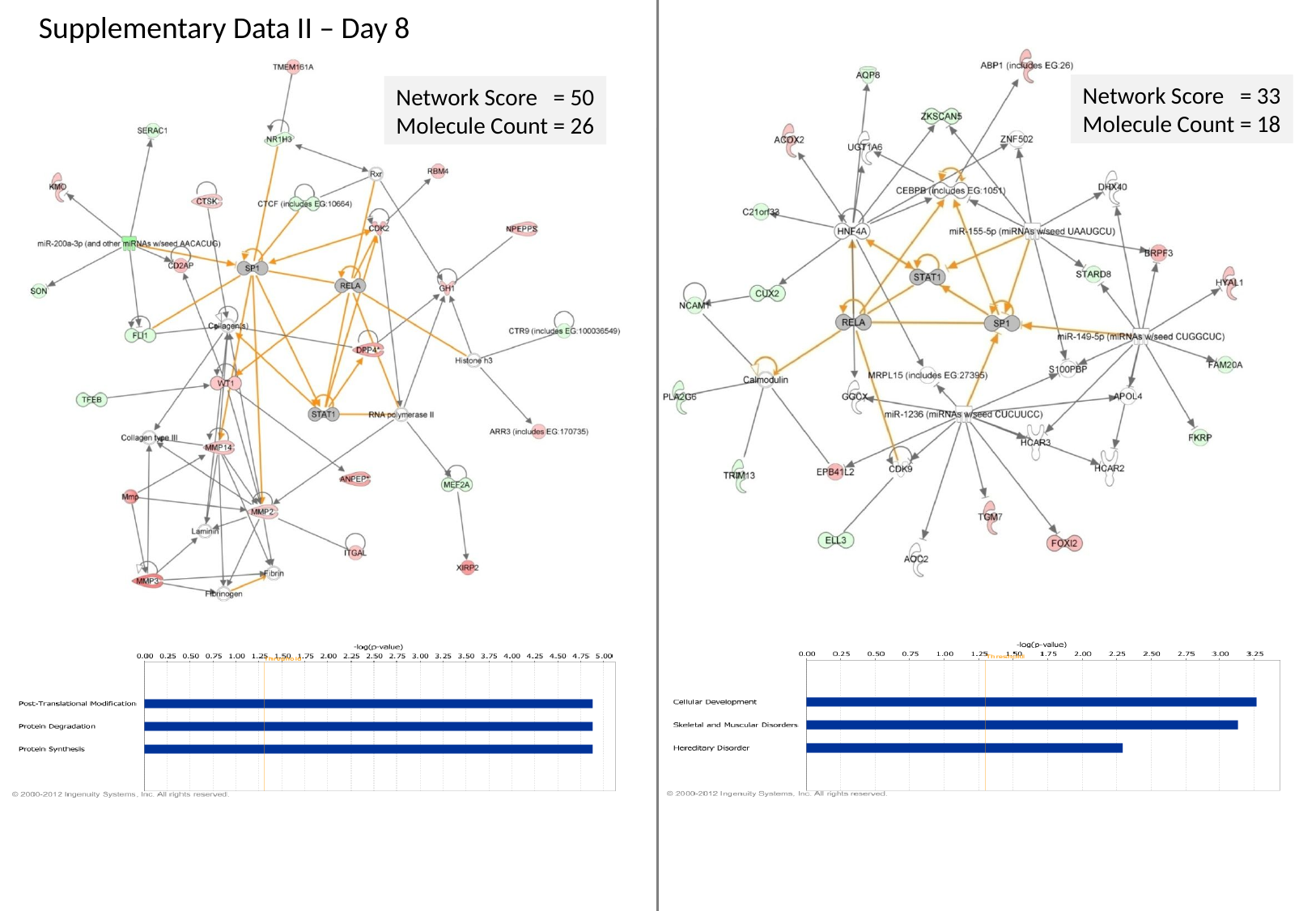

Supplementary Data II – Day 8
Network Score = 33
Molecule Count = 18
Network Score = 50
Molecule Count = 26

## Slide 3
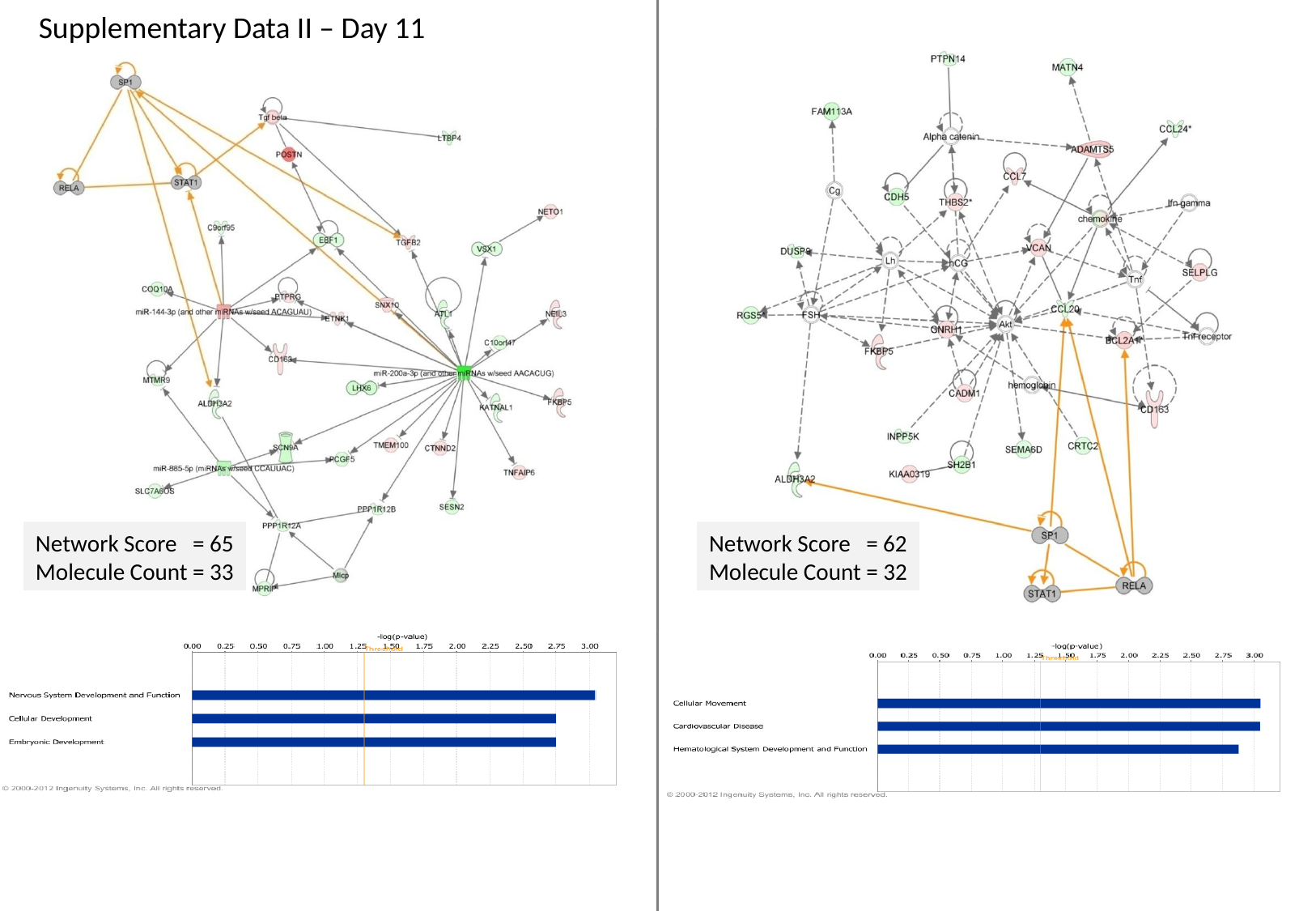

Supplementary Data II – Day 11
Network Score = 65
Molecule Count = 33
Network Score = 62
Molecule Count = 32

## Slide 4
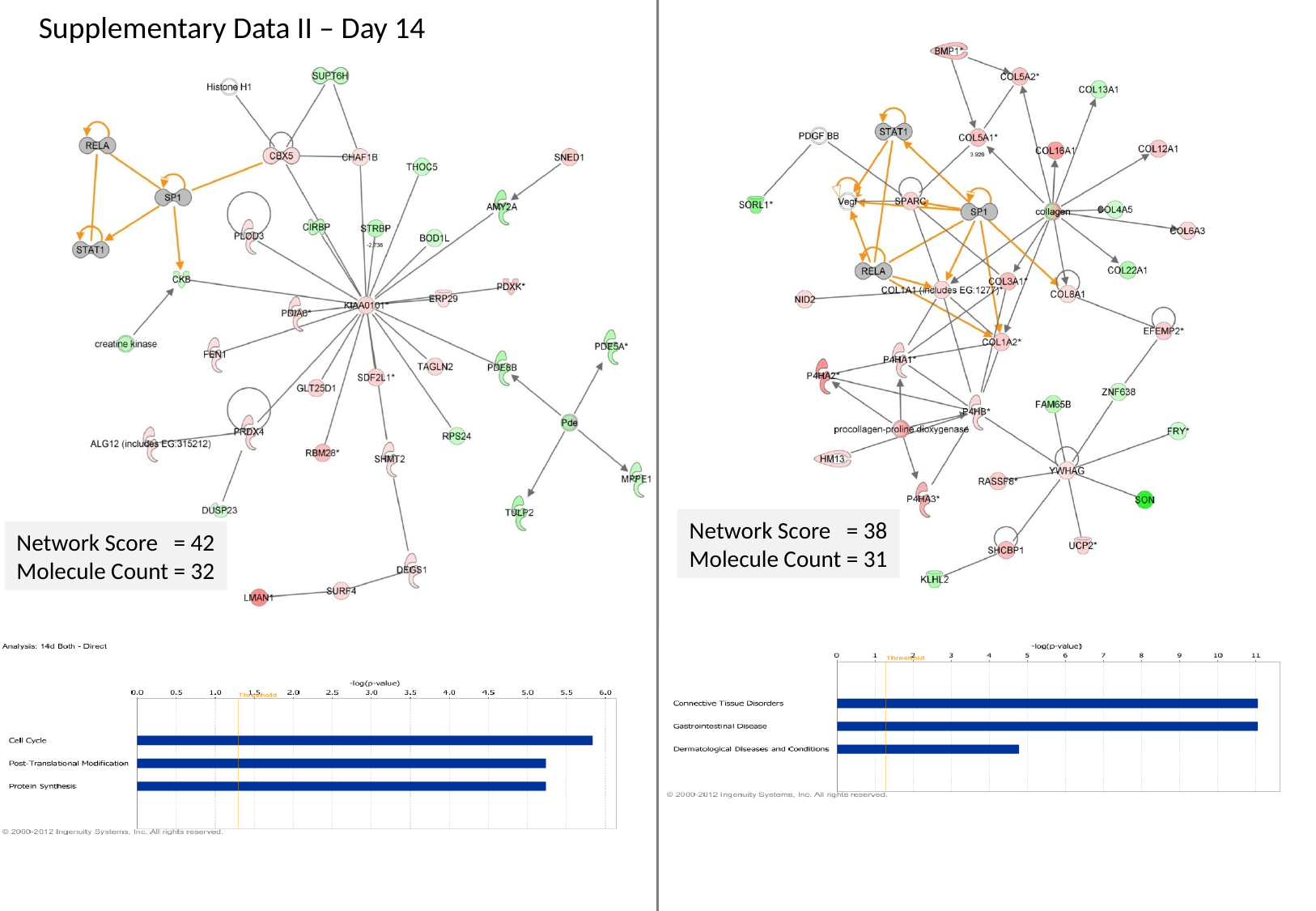

Supplementary Data II – Day 14
Network Score = 38
Molecule Count = 31
Network Score = 42
Molecule Count = 32

## Slide 5
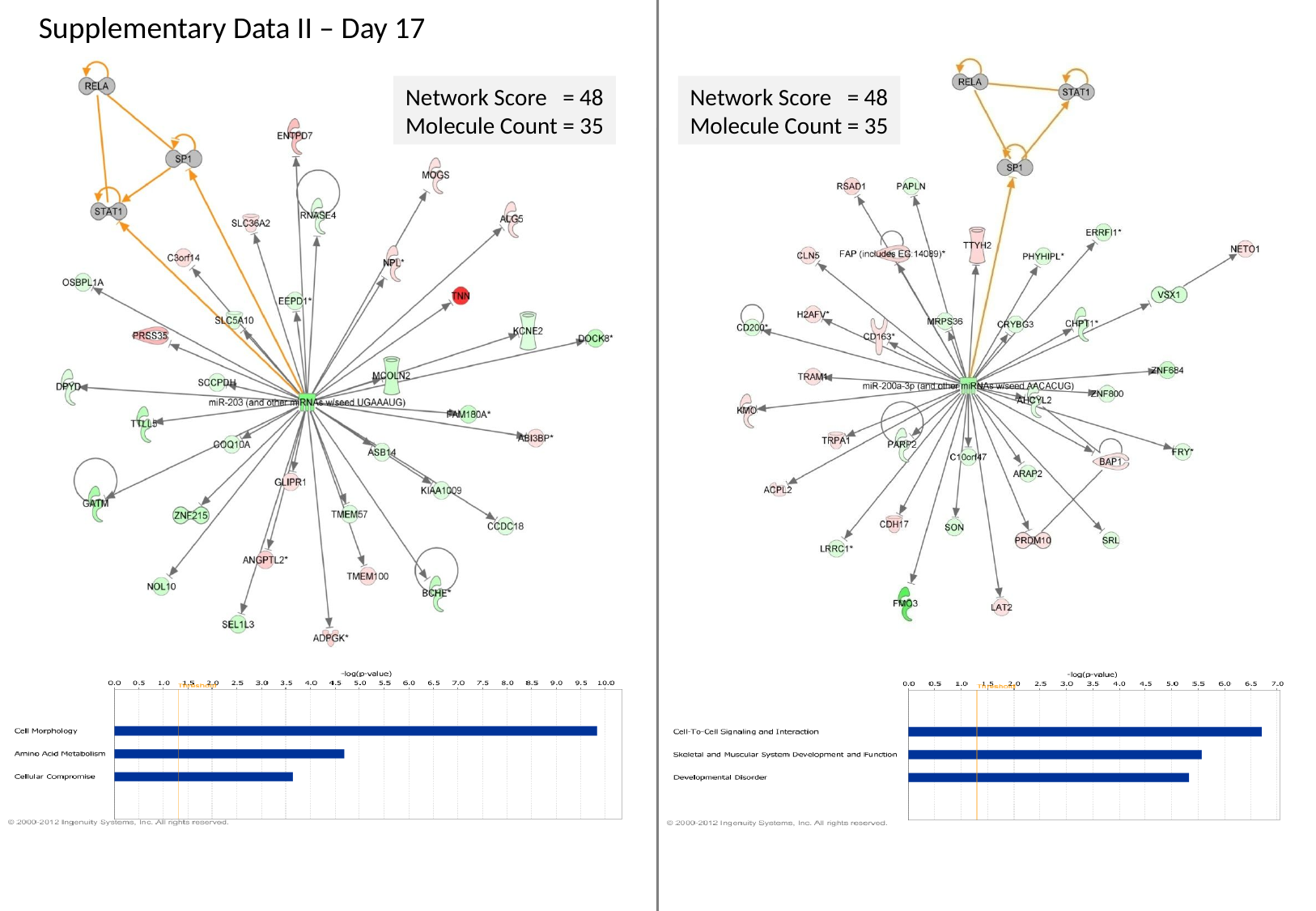

Supplementary Data II – Day 17
Network Score = 48
Molecule Count = 35
Network Score = 48
Molecule Count = 35
